# Anti-Inflammatory and Neurotrophic Factor Production Effects of 3,5,6,7,8,3′,4′-Heptamethoxyflavone in the Hippocampus of Lipopolysaccharide-Induced Inflammation Model Mice

**DOI:** 10.3390/molecules29235559

**Published:** 2024-11-25

**Authors:** Toshiki Omasa, Atsushi Sawamoto, Mitsunari Nakajima, Satoshi Okuyama

**Affiliations:** Department of Pharmaceutical Pharmacology, College of Pharmaceutical Sciences, Matsuyama University, 4-2 Bunkyo-cho, Matsuyama 790-8578, Ehime, Japan; 46210023@g.matsuyama-u.ac.jp (T.O.); mnakajim@g.matsuyama-u.ac.jp (M.N.)

**Keywords:** 3,5,6,7,8,3’4’-heptamethoxyflavone, inflammation, central nervous system, microglia, brain-derived neurotrophic factor, neurogenesis

## Abstract

Citrus fruits contain several bioactive components. Among them, one of the major components is 3,5,6,7,8,3′,4′-heptamethoxyflavone (HMF), which has previously shown protective effects in the brain in some disease models; moreover, HMF has been shown to penetrate the brain. In recent years, inflammation has been identified as a defense response in the body; however, a chronic inflammatory response may trigger several diseases. Inflammation in the peripheral tissues spreads to the brain and is suggested to be closely associated with diseases of the central nervous system. HMF has shown anti-inflammatory effects in the hippocampus following global cerebral ischemia; however, its effects on acute and chronic inflammation in the brain remain unclear. Therefore, in the present study, we examined the effects of HMF in a mouse model of systemic inflammation induced by lipopolysaccharide (LPS) administration. In this study, HMF suppressed LPS-induced microglial activation in the brains of acute inflammation model mice two days after LPS administration. In addition, 24 days after the administration of LPS in a chronic inflammation model, HMF promoted BDNF production and neurogenesis in the brain, which also tended to suppress tau protein phosphorylation at Ser396. These results suggest that HMF has anti-inflammatory and neurotrophic effects in the brains of model mice with lipopolysaccharide-induced systemic inflammation.

## 1. Introduction

The age-related decrease in physiological reserves promotes inflammation in the body [1], which triggers several illnesses [2]. Inflammation is an important response that protects the body from physical stimuli and bacterial and viral invasion and maintains homeostasis in the body. It has also been implicated in various pathological conditions, such as lifestyle-related diseases, autoimmune diseases, cancer, neurodegenerative diseases, and atherosclerosis [3]. However, if this response persists and becomes chronic, it may accelerate the aging of the body and brain [1,4]. Additionally, the prevalence of neurodegenerative diseases is steadily increasing with the aging population; therefore, it is important to proactively adopt preventative and therapeutic approaches for these diseases. One of the substances that induce inflammation is lipopolysaccharide (LPS), an endotoxin produced by Gram-negative bacteria. Intraperitoneal LPS injection induces systemic inflammation that spreads to the brain [5]. Furthermore, it was reported that even when systemic inflammation resolved early, a prolonged inflammation-induced response was observed in the mouse brain for several months, enhancing neuronal damage [6]. Inflammation in the brain has been reported to activate microglia and astrocytes [7], decrease BDNF production [8], inhibit neurogenesis [9,10], and promote the hyperphosphorylation of tau protein [11].

**Figure 1:**
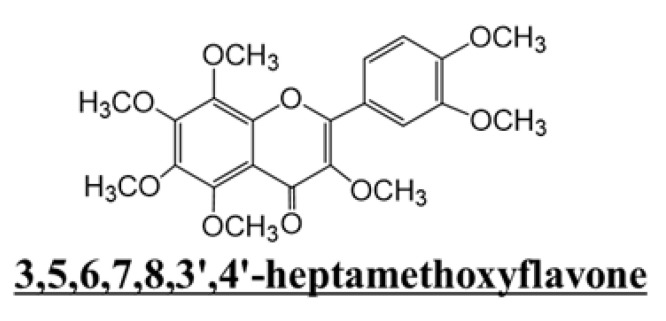
Chemical structure of 3,5,6,7,8,3′,4′-heptamethoxyflavone (HMF).

**Figure 2:**
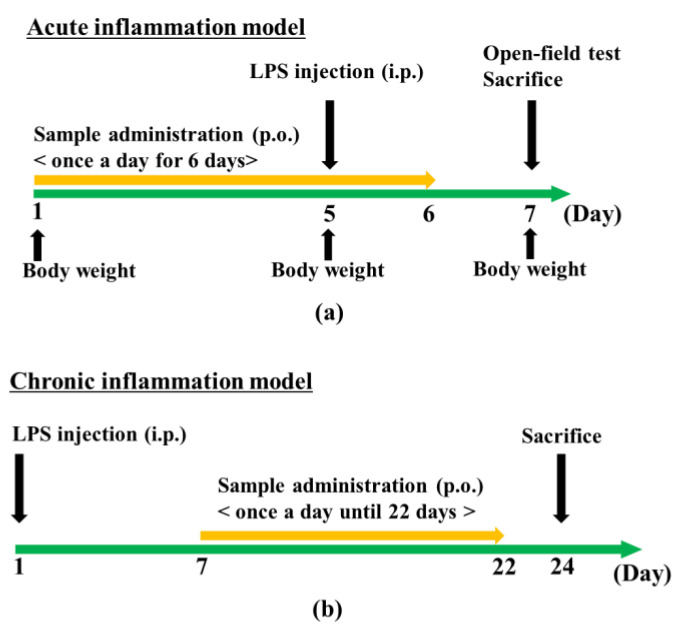
Experimental scheme. (**a**) Acute inflammation model mice were dissected two days after LPS administration. (**b**) Chronic inflammation model mice were dissected 24 days after LPS administration.

Citrus fruits contain various bioactive components, such as polymethoxyflavones, and many studies have focused on their effects on peripheral tissues. Research on the central nervous system (CNS) and citrus fruits has been increasing, and findings have shown that citrus peel components, such as nobiletin and auraptene, improve cognitive function [12,13]. The peel of *Citrus kawachiensis*, a citrus fruit produced in Ehime Prefecture in Japan, contains higher amounts of 3,5,6,7,8,3′,4′-heptamethoxyflavone (HMF; Figure 1) than other citrus fruits [14]. We previously investigated the effects of the subcutaneous administration of HMF in a mouse model of global cerebral ischemia or direct LPS injection into the hippocampus [15]. However, the effect of orally administered HMF on brain inflammation caused by peripheral inflammation has not yet been revealed. Therefore, we examined the effects of the peroral administration of HMF on the brain during acute and chronic inflammation in an LPS-induced systemic inflammation mouse model (Figure 2). In the acute inflammation model mice, we analyzed microglia; on the other hand, in the chronic inflammation model mice, in addition to the analysis of microglia, astrocytes, BDNF, neurogenesis, and hyperphosphorylated tau protein were analyzed to reveal the effects of HMF on the nervous system.

## 2. Results

### 2.1. Acute Inflammation Model

#### 2.1.1. Effects of HMF Administration and LPS Injection on Body Weight

**Figure 3:**
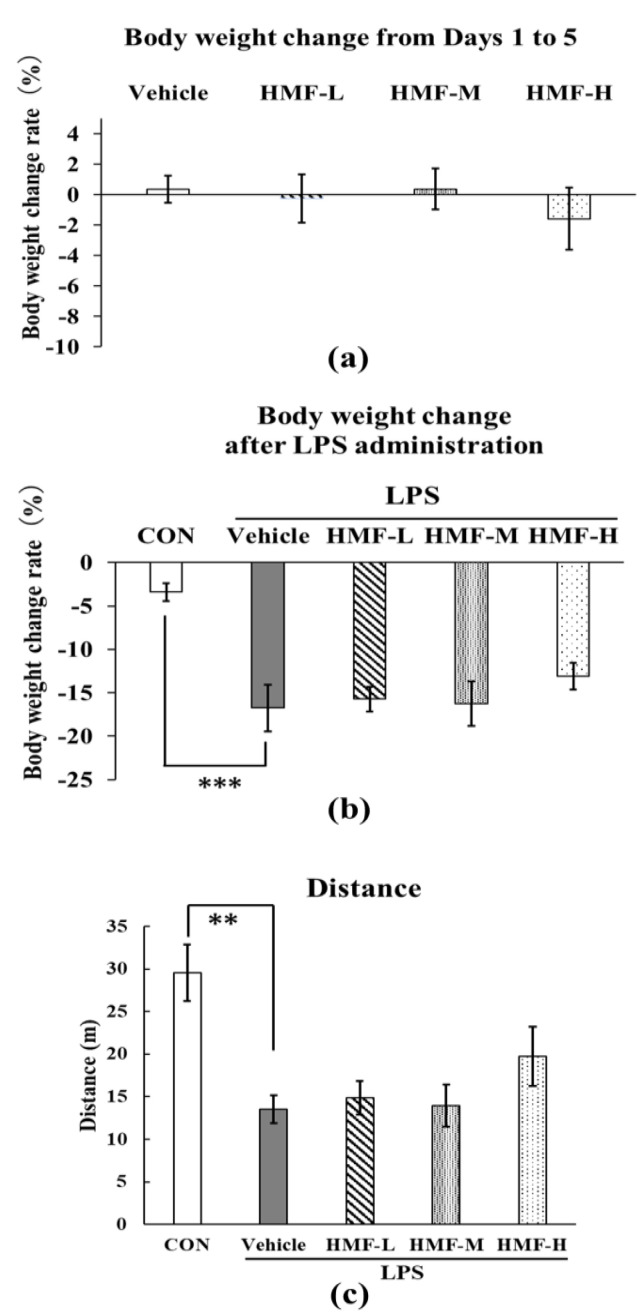
Changes in body weight and measurement of spontaneous locomotive activity after HMF administration and LPS treatment in acute inflammation model mice. Body weight was measured on days 1, 5, and 7. (**a**) Changes in body weight after HMF administration from days 1 to 5. (**b**) Changes in body weight two days after LPS administration. (**c**) Total distance traveled by mice in the open-field test in 10 min. Data were analyzed by performing a one-way ANOVA followed by Dunnett’s multiple comparison test. ** *p* < 0.01 or *** *p* < 0.001 indicates a significant difference between CON and LPS. Values are presented as mean ± SEM (n = 6–8/group).

HMF administration did not significantly affect the body weight of the mice in the four groups from days 1 to 5 (Figure 3a). After LPS administration, body weight was significantly lower in the LPS group than in the CON group (Figure 3b; *** *p* < 0.001). However, HMF administration did not significantly affect weight loss. In addition, LPS-induced inflammation resulted in a significant reduction in locomotor activity in the open-field test in the LPS group compared with that in the CON group, but no significant improvement was observed with the oral administration of HMF (Figure 3c). 

#### 2.1.2. Inhibitory Effect of HMF on Microglial Activation 

**Figure 4:**
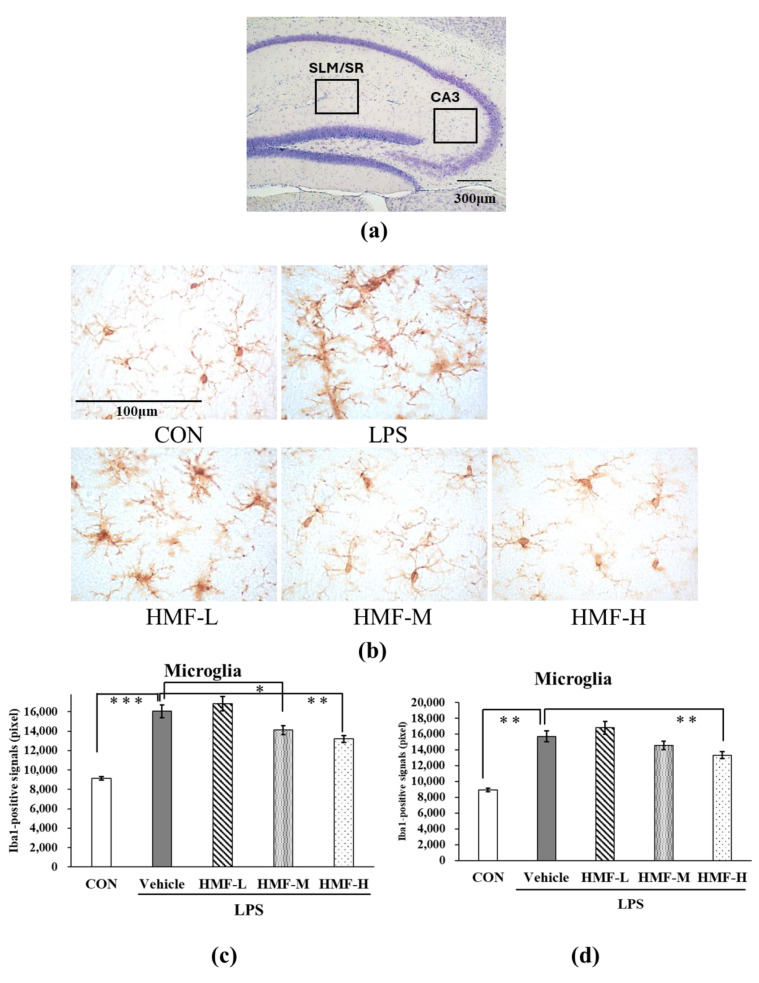
The quantification of activated microglia between the stratum lacunosum moleculare (SLM) and stratum radiatum (SR) and in the CA3 region in the hippocampus of acute inflammation model mice. (**a**) The locations of the captured images and quantification in the hippocampus are shown with squares. (**b**) Representative images of microglia stained with an anti-Iba1 antibody in the SLM and SR. Scale bar = 100 μm. (**c**) The quantification of Iba1-positive signals in the SLM and SR. (**d**) The quantification of Iba1-positive signals in the CA3 region. Data were analyzed by performing a one-way ANOVA followed by Dunnett’s multiple comparison test. ** *p* < 0.01 or *** *p* < 0.001 indicates a significant difference between CON and LPS; * *p* <0.05 indicates a significant difference between LPS and HMF-M; ** *p* < 0.01 indicates a significant difference between LPS and HMF-H. Values are presented as the mean ± SEM (n = 3–4 brain sections/mouse in each group).

Microglia were analyzed in two regions of the hippocampus: between the stratum lacunosum moleculare (SLM) and stratum radiatum (SR) and in the CA3 region. The SLM and SR are areas with many blood vessels in the hippocampus; therefore, microglia were quantified in these areas during acute inflammation. Representative images of the SLM/SR region in the hippocampus are shown in Figure 4b. The Iba1-positive signals were significantly stronger in the LPS group than in the CON group (Figure 4c; *** *p* < 0.001), and this increase was slightly lower in the HMF-M group (Figure 4c; * *p* < 0.05). Furthermore, Iba1-positive signals were significantly weaker in the HMF-H group than in the LPS group (Figure 4c; ** *p* < 0.01). These data demonstrated the dose-dependent effect of HMF. The results of the analysis of microglia in the medial CA3 region were similar to those of the SLM/SR analysis (Figure 4d).

### 2.2. Chronic Inflammation Model

#### 2.2.1. Microglial Activation Following LPS Administration

**Figure 5:**
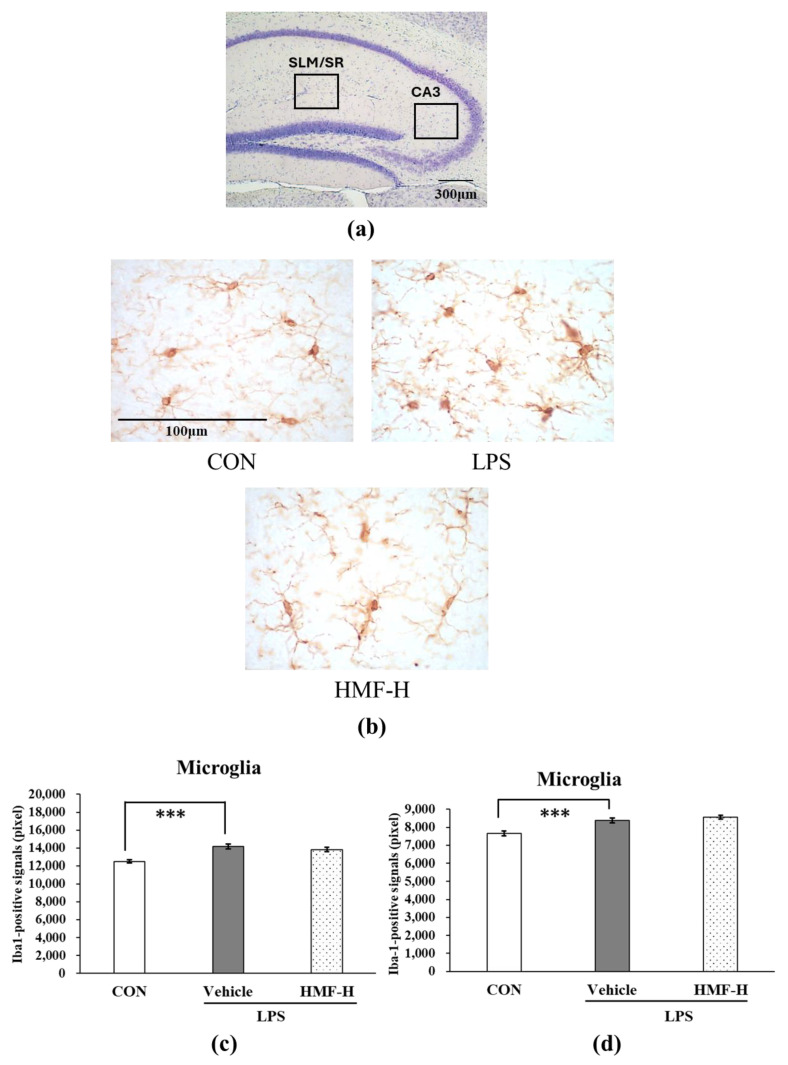
The quantification of activated microglia between the stratum lacunosum moleculare (SLM) and stratum radiatum (SR) and in the CA3 region in the hippocampus of chronic inflammation model mice. (**a**) The locations of the captured images and quantification in the hippocampus are shown with squares. (**b**) Representative microglia pictures stained with an anti-Iba1 antibody in the SLM and SR. The scale bar represents 100 μm. (**c**) The quantification of Iba1-positive signals in the SLM and SR. (**d**) The quantification of Iba1-positive signals in the CA3 region. Data were analyzed by performing a one-way ANOVA followed by Dunnett’s multiple comparison test. *** *p* < 0.001 indicates a significant difference between CON and LPS. Values are presented as the means ± SEMs (n = 3–4 brain sections/mouse in each group).

The HMF-H dose was administered in the chronic inflammation experiment because it was shown to be effective in the acute-phase experiment. Representative images of the SLM/SR in the hippocampus are shown in Figure 5b. The Iba1-positive signals were significantly stronger in the LPS group than in the CON group (Figure 5c; *** *p* < 0.001); however, Iba1-positive signals were not suppressed by HMF-H. The results of the analysis of microglia in the medial CA3 region were similar to those of the SLM/SR analysis (Figure 5d).

#### 2.2.2. HMF Promotes BDNF Production via Astrocyte Activation

**Figure 6:**
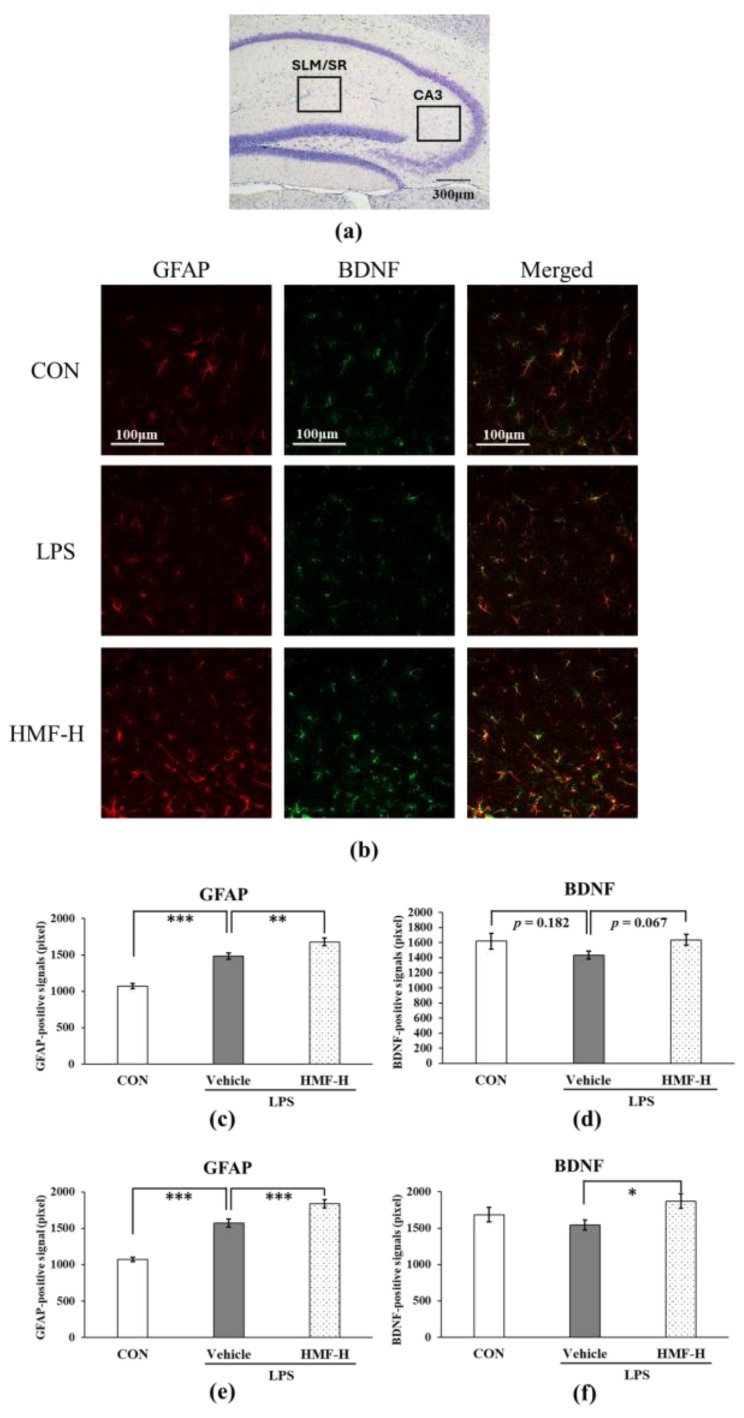
The quantification of astrocytes and BDNF between the stratum lacunosum moleculare (SLM) and stratum radiatum (SR) and in the CA3 region in the hippocampus of chronic inflammation model mice. (**a**) The locations of the captured images and quantification in the hippocampus are shown with squares. (**b**) Representative images of astrocytes, BDNF, and merged, stained with either an anti-GFAP or anti-BDNF antibody in the SLM and SR. Scale bar = 100 μm. (**c**) Quantification of GFAP-positive signals in the SLM and SR. (**d**) The quantification of BDNF-positive signals in the SLM and SR. (**e**) The quantification of GFAP-positive signals in the CA3 region. (**f**) The quantification of BDNF-positive signals in the CA3 region. Data were analyzed by performing a one-way ANOVA followed by Dunnett’s multiple comparison test. *** *p* < 0.001 indicates a significant difference between CON and LPS; * *p* < 0.05, ** *p* < 0.01 or *** *p* < 0.001 indicates a significant difference between LPS and HMF-H. Values are presented as the mean ± SEM (n = 3–4 brain sections/mouse in each group).

Glial fibrillary acidic protein (GFAP) is a well-known marker of astrocytes, and its expression increases with astrocyte activation. The GFAP and BDNF levels were quantified in the hippocampal SLM/SR layers in a mouse model of chronic inflammation. Representative images with each stain from this analysis are shown in Figure 6b. GFAP-positive signals were significantly higher in the LPS group than in the CON group (Figure 6c; *** *p* < 0.001), whereas GFAP-positive signals were significantly higher in the HMF-H group than in the LPS group (Figure 6c; ** *p* < 0.01). The BDNF-positive signals were slightly lower in the LPS group than in the CON group (Figure 6d; *p* = 0.182), even with an increase in GFAP-positive signals in the SLM and SR layers. In contrast, the BDNF-positive signals were higher in the HMF-H group than in the LPS group (Figure 6d; *p* = 0.067). In addition, the quantification of the medial CA3 region in the hippocampus showed a similar increase in GFAP-positive and BDNF-positive signals in the SLM and SR regions in the HMF-H group (Figure 6e,f).

#### 2.2.3. HMF Stimulates Neurogenesis

**Figure 7:**
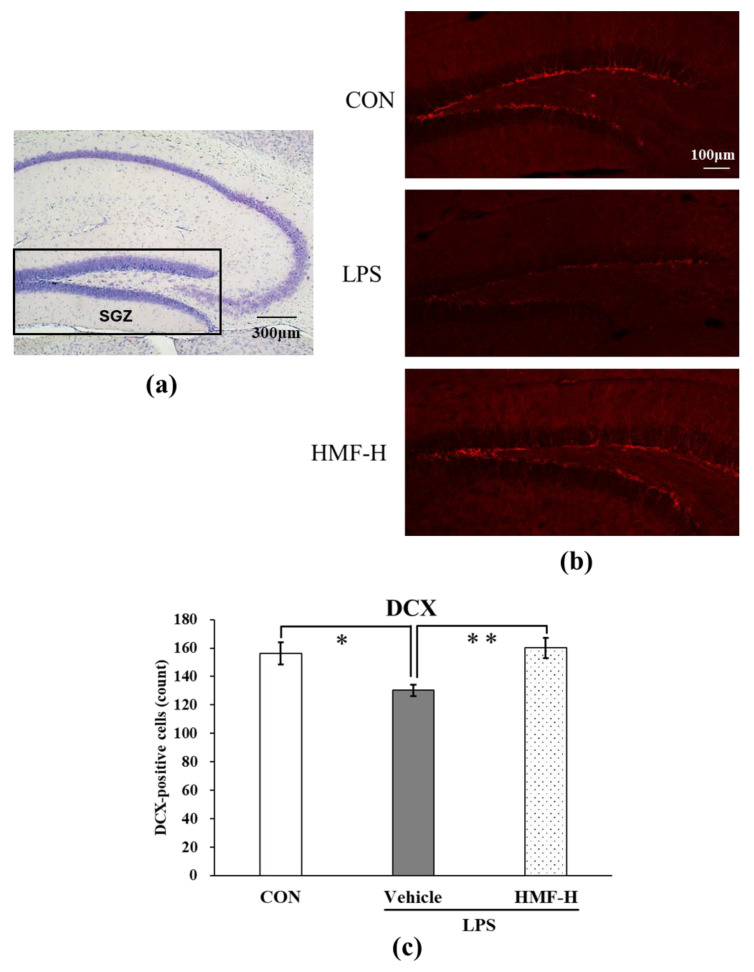
The quantification of DCX in the subgranular zone (SGZ) of the hippocampus of chronic inflammation model mice. (**a**) The location of the captured images and quantification in the hippocampus is shown with a square. (**b**) Representative images of DCX stained with anti-DCX antibody. Scale bar = 100 μm. (**c**) The quantification of DCX-positive cells with nuclei in the SGZ of the hippocampus. Data were analyzed by performing a one-way ANOVA followed by Dunnett’s multiple comparison test. * *p* < 0.05 indicates a significant difference between CON and LPS; ** *p* < 0.01 indicates a significant difference between LPS and HMF-H. Values are represented as the mean ± SEM (n = 2 brain sections/mouse in each group).

Doublecortin (DCX), a marker of neurogenesis, is highly expressed in neural progenitor cells, and it induces the differentiation of cells into neurons. The DCX-positive cells with nuclei in the subgranular zone of the dentate gyrus (SGZ) were manually counted and quantified. Representative images from this analysis are shown in Figure 7b. The number of DCX-positive cells was significantly lower in the LPS group than in the CON group (Figure 7c; * *p* < 0.05), whereas it was significantly higher in the HMF-H group than in the LPS group (Figure 7c; ** *p* < 0.01).

#### 2.2.4. HMF Suppresses Tau Phosphorylation

**Figure 8:**
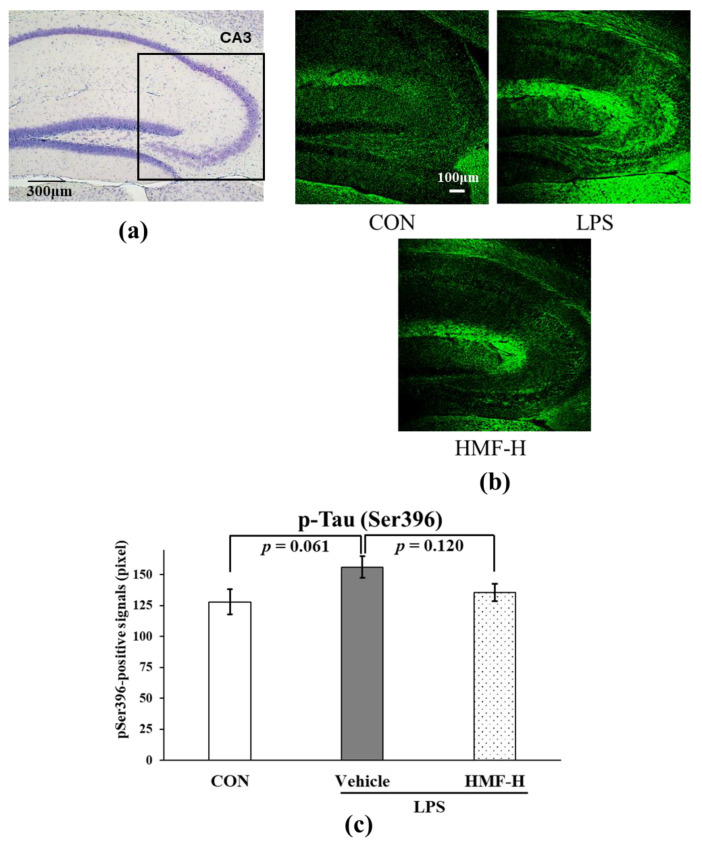
The quantification of phosphorylated tau protein at Ser396 in the CA3 region of the hippocampus of chronic inflammation model mice. (**a**) The location of the captured images and quantification in the hippocampus is shown with a square. (**b**) Representative images of pSer396 stained with anti-pSer396 antibody. Scale bar = 100 μm. (**c**) Immunopositive signals of pSer396 in the medial hippocampal CA3 region were quantified. Data were analyzed by performing a one-way ANOVA followed by Dunnett’s multiple comparison test. Values are presented as the mean ± SEM (n = 3–4 brain sections/mouse in each group).

We quantified tau phosphorylation at the serine 396 residue in the medial CA3 region. Representative images of this region are shown in Figure 8b. Phosphorylated Ser396-positive signals were more pronounced in the LPS group than in the CON group (Figure 8c; *p* = 0.061), and this increase was suppressed in the HMF-H group (Figure 8c; *p* = 0.120).

## 3. Discussion

Inflammatory cytokines in the serum were previously shown to increase within a few hours via the activation of white blood cells, such as neutrophils and monocytes, after the induction of inflammation via intraperitoneal administration of LPS. These cells penetrate the brain by passing through the blood–brain barrier and activate microglia [5,16,17]. Activated microglia release inflammatory cytokines in the brain, which spread inflammation and ultimately lead to neuronal cell death [6]. Toll-like receptors (TLRs) are common receptors mainly found in the cells, including macrophages and dendritic cells, of the innate immune system, which recognize microbial components, as well as LPS, and play an important role in the immune response [18]. The decreases in body weight and locomotive behavior observed in this experiment after LPS administration suggested that LPS induced inflammation; however, HMF did not produce any improvement with this dosage schedule (Figure 3).

The activation of microglia, which are immune-responsive cells in the brain, is an indicator of inflammation in the CNS. In addition, many blood vessels are located around the SLM and SR in the hippocampus, where immunoreactivity is observed following LPS treatment [19]. Microglia possess multiple branches with ramified processes in the normal state. However, microglia are known to acquire an amoeboid shape in an activated state when induced by inflammatory stimuli [20]. The quantification of activated microglia in the mouse model of acute inflammation confirmed that inflammation spread to the brain as microglia were activated following LPS administration. In contrast, the peroral administration of HMF suppressed the activated microglia (Figure 4), similar to the results previously reported with the subcutaneous administration of HMF and intrahippocampal LPS injection, suggesting that HMF can suppress inflammation in the brain [15]. Furthermore, we previously demonstrated that HMF can penetrate the brain, and the concentration-dependent effects observed in the present study suggest a correlation between the concentration of HMF, its transport to the brain, and its ability to inhibit microglial activation [21,22]. The mechanisms underlying the anti-inflammatory effects of HMF remain unclear; however, we previously reported that HMF inhibits PDE-4 [23], which can subsequently activate the signaling pathways that suppress high mobility group box 1 (HMGB1) by increasing cAMP levels [24,25,26]. TLR4, which is expressed in the microglia and astrocytes in the brain, recognizes HMGB1 and is involved in the release of inflammatory mediators through the activation of the NF-kB signaling pathway [18,24]. In future studies, we plan to examine the characteristics and mechanisms of action of HMF in cultured microglia.

In the chronic inflammation mouse model, sustained microglial activation was observed following LPS administration (Figure 5), which is similar to previously reported findings [6,17]. This result may be due to the prolonged activation of the microglia in the LPS group because chronic inflammation is long-term inflammation; in contrast, the HMF-H group did not show any difference. Microglia are generally classified as exhibiting inflammatory (M1) or neuroprotective (M2) activity [27], and the HMF-H group showed neuroprotective results in this experiment, suggesting that the M2 type may have been increased. Anti-Iba1 antibodies can stain microglia but cannot differentiate between the M1 and M2 types. Future work to distinguish between the M1 and M2 types should further clarify the effects of HMF on microglia. It has been reported that microglia activated by inflammation activate astrocytes and decrease BDNF production [7,8]. In this study, although astrocytes were activated in the LPS group, and BDNF levels tended to decrease, both astrocyte activation and BDNF expression increased in the HMF-H group (Figure 6). These results could also be attributed to the phenotypic differences in astrocytes. Astrocytes are categorized into those with inflammatory (A1) and protective (A2) properties [28]. A2 astrocytes play a neuroprotective role by enhancing the production of BDNF [29]. Therefore, the glial cell activation in the HMF-H group may support the protective inclination of glial cells in the brain with a similar mechanism to that in our previous in vitro experiment [23]. DCX is a marker of neurogenesis, and the SGZ is one of the regions where neurogenesis is known to occur [30]. Neurogenesis in the hippocampal SGZ plays an important role in learning memory and has been shown to be suppressed in LPS-induced neuroinflammation model mice and depressive-like behavior model mice in previous studies [9,10]. In the present study, a significant decrease in DCX expression was observed in the SGZ of chronic inflammation model mice in the LPS group, whereas an increase was observed in the HMF-H group (Figure 7). This result correlated with BDNF expression; increased BDNF levels enhanced neurogenesis [31]. These findings suggest that inflammation-stimulated astrocytes induce prolonged inflammation by increasing the number of A1 astrocytes, but HMF protects the brain by increasing the proportion of A2 astrocytes [28,29]. The phenotypes and characteristics of glial cells should be carefully investigated in future studies. 

Recently, chronic inflammation has been identified as a risk factor for Alzheimer’s disease [11,27,32,33]. One of the characteristic pathological findings of Alzheimer’s disease is the presence of neurofibrillary tangles, which are induced by excessive tau protein phosphorylation at several sites [33,34]. Again, inflammation is one of the factors that promotes the phosphorylation of tau protein [35]. In the present study, in the HMF-H group, the hyperphosphorylation of tau induced by chronic inflammation tended to be suppressed (Figure 8), which may be a preventive strategy against Alzheimer’s disease [36]. However, we could only demonstrate serine 396 phosphorylation; therefore, further experiments are needed.

In mice with peripheral inflammation, the oral administration of HMF suppressed microglial activation in the brain during the acute phase; moreover, HMF promoted BDNF production and neurogenesis in the brain during chronic inflammation. These results indicate that HMF has the potential to prevent various inflammation-related diseases in the brain.

## 4. Materials and Methods

### 4.1. Animals

Nine-week-old C57BL/6N male mice were purchased from Japan SLC (Hamamatsu, Japan). Mice were kept at 23 ± 1 °C under a 12 h light/dark cycle (light period 8:00–20:00, dark period 20:00–8:00). Mice had free access to water and food during the experimental period. All animal experiments were performed according to the regulations of the Matsuyama University Animal Experiment Committee (approval numbers: #21-013, #22-011, #23-012).

### 4.2. Experimental Design

HMF (Figure 1) was obtained from Ushio ChemiX Corporation (Omaezaki, Japan), and LPS was purchased from Sigma-Aldrich (*Salmonella enterica* serotype Typhimurium; St. Louis, MO, USA). In the present study, two experimental groups were established: the acute inflammation group, which was dissected two days after LPS administration (Figure 2a and Table 1), and the chronic inflammation group, which was dissected 24 days after LPS administration (Figure 2b and Table 1). 

The acute inflammation model consisted of a control (CON) group, an LPS group, an HMF low-dose (HMF-L) group that received 50 mg/kg of HMF and LPS, an HMF mid-dose (HMF-M) group that received 100 mg/kg of HMF and LPS, and an HMF high-dose (HMF-H) group that received 300 mg/kg of HMF and LPS. HMF was dissolved in a mixture of 10% dimethyl sulfoxide and 10% Tween 20 in water and administered orally once daily for six days. Both the CON and LPS groups received vehicle solutions. LPS was dissolved in saline at a concentration of 2 mg/kg and administered intraperitoneally as a single injection on the fifth day of HMF administration. 

A chronic inflammation model was established with CON, LPS, and HMF-H groups. HMF was prepared as described above and orally administered once daily for 16 days. LPS was dissolved in saline at a dose of 5 mg/kg and administered intraperitoneally as a single injection one week prior to HMF administration.

### 4.3. Open-Field Test

In the acute and chronic inflammation phase models, mice were placed in a 70 × 70 × 50 cm box-shaped open-field device and allowed to explore freely for 10 min. The distance traveled and immobility time during the 10 min period were analyzed using an ANY-maze Video Tracking System (Stoelting, Wood Dale, IL, USA) connected to a USB digital camera to evaluate the spontaneous behavior of the mice. Measurements were performed under indirect illumination in the dark. Feces and urine were removed after each test, and the inside of the apparatus was kept clean by means of alcohol disinfection.

### 4.4. Immunohistochemistry for Optical Microscopy

In each experiment, the mice were sacrificed through brief exposure to isoflurane. Blood was drawn from the heart, and the mice were perfused transcardially with 25 mL of ice-cold phosphate-buffered saline (PBS). The brains were rapidly removed, immersed in freshly depolymerized 4% paraformaldehyde for 48 h, and cryoprotected via successive 24 h immersion in 15% and 30% sucrose in PBS immediately before sectioning. Fixed and frozen brain sections were cut at a thickness of 30 µm (sagittal plane) using a cryostat (CM3050; Leica Microsystems, Heidelberger, Germany). Sagittal brain sections 1.0 mm to 2.0 mm lateral from the brain midline were stained using the following tissue-staining protocols.

Endogenous peroxidase was inactivated by immersing the sections in 3% hydrogen peroxide for 20 min. The sections were blocked with 2% skim milk for 30 min, followed by 5% normal goat serum solution for 1 h, and then incubated with each primary antibody at 4 °C overnight (Table 2). The following day, the sections were soaked in secondary antibodies for 1 h, mounted on glass slides, and stained with 3,3’-diaminobenzidine (DAB). 

Glass slides were soaked in 95% ethanol, 100% ethanol, and xylene and then sealed with a cover glass. Images of the stained brain sections were captured using an optical microscope, and the immune-positive signals obtained using ImageJ 1.48v software (NIH, Bethesda, MD, USA) were quantified and analyzed.

### 4.5. Immunofluorescence for Confocal Microscopy

Frozen sagittal brain sections were defrosted in PBS and immersed in HistoVT One (NACALAI TESQUE, Kyoto, Japan) at 70 °C for 20 min for antigen retrieval. The sections were incubated at room temperature, blocked with 2% skim milk for 30 min, followed by incubation in 5% normal goat serum solution for 1 h, and then immersed in primary antibodies at 4 °C overnight (Table 2). The following day, the sections were immersed in suitable secondary antibodies and shaken for 1 h in the dark. Brain sections were placed on glass slides and covered with a mounting medium containing DAPI (Vectashield; Vector Laboratories, Burlingame, CA, USA). Images were captured using a confocal fluorescence microscope (LSM800; Zeiss, Oberkochen, Germany) and quantified, and immunofluorescence signals were analyzed using ImageJ 1.48v software (NIH).

### 4.6. Statistical Analysis

Data for individual groups are shown as the mean ± SEM. Statistical analyses were performed using a one-way ANOVA followed by Dunnett’s multiple comparison test among groups of acute inflammation model mice or chronic inflammation model mice. Statistical significance was set at *p* < 0.05.

## Figures and Tables

**Table 1:** Categorization of animal groups in this study and their treatments.

Group	n	Sample (p.o.)	Treatment (i.p.)
**Acute Inflammation Model**			
CON	8	Vehicle (10% DMSO + 10% Tween20)	Saline
LPS	8	Vehicle	LPS (2 mg/kg)
HMF-L	8	Vehicle + HMF (50 mg/kg)	LPS
HMF-M	8	Vehicle + HMF (50 mg/kg)	LPS
HMF-H	8	Vehicle + HMF (50 mg/kg)	LPS
**Chronic Inflammation Model**			
CON	8	Vehicle	Saline
LPS	15	Vehicle	LPS (5 mg/kg)
HMF-H	15	Vehicle + HMF (50 mg/kg)	LPS

**Table 2:** Primary antibodies used for immunohistochemical staining and immunofluorescence.

Antibody	Epitope Protein/Amino Acid	Host	Dilution	Resource
Iba1	Ionized calcium binding adaptor molecule1	Rabbit	1:1000	Wako, Osaka, Japan
GFAP	Glial fibrillary acid protein	Mouse	1:500	Sigma-Aldrich, St. Louis, MO, USA
BDNF	Brain-derived neurotrophic factor	Rabbit	1:250	Abcam, Cambridge, UK
p-Ser396	Phosphorylated-tau at Serine 396	Rabbit	1:500	AnaSpec, Fremont, CA, USA
DCX	Doublecortin	Rabbit	1:800	Cell signaling, Danvers, MA, USA

## Data Availability

Data are contained within this article.

## References

[1] Chen X., Mao G., Leng S.X. (2014). Frailty syndrome: An overview. Clin. Interv. Aging.

[2] Bektas A., Schurman S.H., Sen R., Ferrucci L. (2018). Aging, Inflammation and the environment. Exp. Gerontol..

[3] Mou Y., Du Y., Zhou L., Yue J., Hu X., Liu Y., Chen S., Lin X., Zhang G., Xiao H. (2022). Gut microbiota interact with the brain through systemic chronic inflammation: Implications on neuroinflammation, neurodegeneration, and aging. Front. Immunol..

[4] Stephenson J., Nutma E., van der Valk P., Amor S. (2018). Inflammation in CNS neurodegenerative diseases. Immunology.

[5] Huang X., Hussain B., Chang J. (2021). Peripheral inflammation and blood-brain barrier disruption: Effects and mechanisms. CNS Neurosci. Ther..

[6] Qin L., Wu X., Block M.L., Liu Y., Breese G.R., Hong J.S., Knapp D.J., Crews F.T. (2007). Systemic LPS causes chronic neuroinflammation and progressive neurodegeneration. Glia.

[7] Li B., Du L., Wu S., Yin Y. (2024). Protective effects of taurine on heat stress-induced cognitive impairment through Npas4 and Lcn2. Int. Immunopharmacol..

[8] El-ezz D.A., Aldahmash W., Esatbeyoglu T., Afifi S.M., Elbaset M.A. (2024). Cilostazol combats lipopolysaccharide-induced hippocampal injury in rats: Role of AKT/GSK3b/CREB curbing neuroinflammation. Adv. Pharmacol. Pharm. Sci..

[9] Pérez-Domínguez M., Ávila-Muñoz E., Domínguez-Rivas E., Zepeda A. (2019). The detrimental effects of lipopolysaccharide-induced neuroinflammation on adult hippocampal neurogenesis depend on the duration of the pro-inflammatory response. Neural Regen. Res..

[10] Liu L., Zhang Q., Cai Y., Sun D., He X., Wang L., Yu D., Li X., Xiong X., Xu H. (2016). Resveratrol counteracts lipopolysaccharide-induced depressive-like behaviors via enhanced hippocampal neurogenesis. Oncotarget.

[11] Roe A.D., Staup M.A., Serrats J., Sawchenko P.E., Rissman R.A. (2011). Lipopolysaccharide-induced tau phosphorylation and kinase activity-modulation, but not mediation, by corticotropin-releasing factor receptors. Eur. J. Neurosci..

[12] Nakajima A., Ohizumi Y., Yamada K. (2014). Anti-dementia activity of nobiletin, a citrus flavonoid: A review of animal studies. Clin. Psychopharmacol. Neurosci..

[13] Qi G., Mi Y., Fan R., Li R., Liu Z., Liu X. (2019). Nobiletin protects against systemic inflammation-stimulated memory impairment via MAPK and NF-κB signaling pathways. J. Agric. Food Chem..

[14] Amakura Y., Yoshimura M., Ouchi K., Okuyama S., Furukawa Y., Yoshida T. (2013). Characterization of constituents in the peel of *Citrus kawachiensis* (Kawachibankan). Biosci. Biotechnol. Biochem..

[15] Okuyama S., Miyoshi K., Tsumura Y., Amakura Y., Yoshimura M., Yoshida T., Nakajima M., Furukawa Y. (2015). 3.5.6.7.8,3’,4’-heptamethoxyflavone, a citrus polymethoxylated flavone, attenuates inflammation in the mouse hippocampus. Brain Sci..

[16] Biesmans S., Meert T.F., Bouwknecht J.A., Acton P.D., Davoodi N., Haes P.D., Kuijlaars J., Langlois X., Matthews L.J.R., Donck L.V. (2013). Systemic immune activation leads to neuroinflammation and sickness behavior in mice. Mediat. Inflamm..

[17] Liu B., Hong J.S. (2003). Role of microglia in inflammation-mediated neurodegenerative diseases: Mechanisms and strategies for therapeutic intervention. J. Pharmacol. Exp. Ther..

[18] Muhammad T., Ikram M., Ullah R., Rehman S.U., Kim M.O. (2019). Hesperetin, a citrus flavonoid, attenuates LPS-induced neuroinflammation, apoptosis and memory impairments by modulating TLR4/NF-κB signaling. Nutrients.

[19] Chung D.W., Yoo K.Y., Hwang I.K., Kim D.W., Chung J.Y., Lee C.H., Choi J.H., Choi S.Y., Youn H.Y., Lee I.S. (2010). Systemic administration of lipopolysaccharide induces cyclooxygenase-2 immunoreactivity in endothelium and increases microglia in the mouse hippocampus. Cell Mol. Neurobiol..

[20] Nakamura Y. (2002). Regulating factors for microglial activation. Biol. Pharm. Bull..

[21] Okuyama S., Miyazaki K., Yamada R., Amakura Y., Yoshimura M., Sawamoto A., Nakajima M., Furukawa Y. (2017). Permeation of polymethoxyflavones into the mouse brain and their effect on MK-801-induced locomotive hyperactivity. Int. J. Mol. Sci..

[22] Sawamoto A., Okuyama S., Amakura Y., Yoshimura M., Yamada T., Yokogoshi H., Nakajima M., Furukawa Y. (2017). 3,5,6,7,8,3′,4′-heptamethoxyflavone ameliorates depressive-like behavior and hippocampal neurochemical changes in chronic unpredictable mild stressed mice by regulating the brain-derived neurotrophic factor: Requirement for ERK activation. Int. J. Mol. Sci..

[23] Sawamoto A., Okuyama S., Nakajima M., Furukawa Y. (2019). Citrus flavonoid 3,5,6,7,8,3′,4′-heptamethoxyflavone induces BDNF via cAMP/ERK/CREB signaling and reduces phosphodiesterase activity in C6 cells. Pharmacol. Rep..

[24] Gerö D., Szoleczky P., Módis K., Pribis J.P., Al-Abed Y., Yang H., Chevan S., Billiar T.R., Tracey K.J., Szabo C. (2013). Identification of pharmacological modulators of HMGB1-induced inflammatory response by cell-based screening. PLoS ONE.

[25] Wang Y., Zhang T., Zhao H., Qi C., Ji X., Yan H., Cui R., Zhang G., Kang Y., Shi G. (2021). Pentoxifylline enhances antioxidative capability and promotes mitochondrial biogenesis in D-galactose-induced aging mice by increasing Nrf2 and PGC-1α through the cAMP-CREB pathway. Oxid. Med. Cell Longev..

[26] El-Sadek H.M., AL-Shorbagy M.Y., Awny M.M., Abdallah D.M., El-Abhar H.S. (2021). Pentoxifylline treatment alleviates kidney ischemia/reperfusion injury: Novel involvement of galectin-3 and ASK-1/JNK & ERK1/2/NF-κB/HMGB-1 trajectories. J. Pharmacol. Sci..

[27] Guo S., Wang H., Yin Y. (2022). Microglia polarization from M1 to M2 in neurodegenerative diseases. Front. Aging Neurosci..

[28] Fan Y.Y., Huo J. (2021). A1/A2 astrocytes in central nervous system injuries and diseases: Angels or devils?. Neurochem. Int..

[29] Qiao Y., Liu H., He C., Ma Y. (2024). ApoE Mimic Peptide COG1410 Reduces Aβ Deposition and Improves Cognitive Function by Inducing the Transformation of A1/A2 Reactive Astrocytes and Increasing the BDNF Concentration in Brain of APP/PS1 Double Transgenic Mice. Neurosci..

[30] Guo J., Yu C., Li H., Liu F., Feng R., Wang H., Meng Y., Li Z., Ju G., Wang J. (2010). Impaired neural stem/progenitor cell proliferation in streptozotocin-induced and spontaneous diabetic mice. Neurosci. Res..

[31] Liu H., Xue X., Shi H., Qi L., Gong D. (2015). Osthole upregulates BDNF to enhance adult hippocampal neurogenesis in APP/PS1 transgenic mice. Biol. Pharm. Bull..

[32] Zakaria R., Wan Yaacob W.M.W., Othman Z., Long I., Ahmad A.H., Al-Rahbi B. (2017). Lipopolysaccharide-induced memory impairment in rats: A model of Alzheimer’s disease. Physiol. Res..

[33] Metcalfe M.J., Figueiredo-Pereira M.E. (2010). Relationship between tau pathology and neuroinflammation in Alzheimer’s disease. Mt. Sinai, J. Med..

[34] Sy M., Kitazawa M., Medeiros R., Whitman L., Cheng D., Lane T.E., LaFerla F.M. (2011). Inflammation induced by infection potentiates tau pathological features in transgenic mice. Am. J. Pathol..

[35] Kamat P.K. (2015). Streptozotocin induced Alzheimer’s disease like changes and the underlying neural degeneration and regeneration mechanism. Neural Regen. Res..

[36] Medeiros R., Baglietto-Vargas D., LaFerla F.M. (2011). The role of tau in Alzheimer’s disease and related disorders. CNS Neurosci. Ther..

